# Insights Into Mechanisms of Tumor and Immune System Interaction: Association With Wound Healing

**DOI:** 10.3389/fonc.2019.01115

**Published:** 2019-10-25

**Authors:** Aleksandr V. Ponomarev, Irina Zh. Shubina

**Affiliations:** N.N. Blokhin National Medical Research Center of Oncology, Moscow, Russia

**Keywords:** monocytes, macrophages, hypothesis, inflammation, growth factors, immunosuppression, tumor, wound healing

## Abstract

A large number of studies have presented a great deal of information about tumor and immune system interaction. Nevertheless, the problem of tumor evasion from the immune reaction is still difficult to resolve. Understanding the ways in which immunosuppressive tumor microenvironment develops and maintains its potential is of utmost importance to ensure the best use of the suppressed immune functions. The study presents a review covering the data on tumor-associated antigens, mechanisms of tumor evasion from the immune reactions, and search for common immunosuppressive processes of tumor growth and normal wound healing. The study discusses the important role of monocytes/macrophages in the regulation of immune system reactions. We suggest that the simultaneous actions of growth factors and pro-inflammatory cytokines may result in the suppression of the immune system. The study describes intracellular signaling molecules that take part in the regulation of the myeloid cell functions. If the hypothesis is proved correct, the indicated interaction of cytokines could be regarded as a prospective target for antitumor therapy.

## Introduction

The immune system can recognize malignantly transformed cells due to the antigens that differentiate a tumor cell from the normal one. Inflammation in the tumor microenvironment causes an accumulation of immune cells at the site. Therefore, the tumor has some mechanisms of immune suppression in the microenvironment to evade immune surveillance. Besides, an obscure inflammatory phenomenon associated with immunosuppression has been observed. Immunosuppression requires no new mechanisms of action in the tumor microenvironment, but it boosts existing normal regulatory mechanisms, such as those that participate in inflammation resolution, wound healing, etc. Better understanding of these mechanisms is crucially important. The paper provides evidence that the concurrent presence of pro-inflammatory cytokines and growth factors affecting monocytes/macrophages in the tumor microenvironment may act as such a regulatory mechanism. This combination of cytokines and growth factors can have a significant immunosuppressive effect.

## Mechanisms of the Immunological Recognition of Tumors

Immune cells can act against tumors in different ways, such as by absorbing and presenting tumor antigens, releasing cytokines that activate and recruit other immune cells, or directly killing tumor cells. This section describes the most well-studied tumor antigens that distinguish a tumor cell from a normal one, which help the immune system eventually eliminate the tumor. Some immune mechanisms, such as phagocytosis, involve the recognition and elimination of apoptotic and stressed cells. Many cell types have a special function of efferocytosis, i.e., elimination of apoptotic cells. They include both professional (macrophages and immature dendritic cells) and non-professional phagocytes (fibroblasts and epithelial cells).

### Phosphatidylserines

Phosphatidylserines are phospholipid components located on the inner (cytosolic) cell membranes. In apoptotic cells, phosphatidylserines come out on the cell surface. As a result, phagocytes receive the signal for the absorption of the apoptotic cells. Phosphatidylserine can be recognized by a number of receptors (1, 2). Some studies showed that tumor cells may have an increased level of surface phosphatidylserines (3).

### Calreticulin

Another pro-phagocyte signal is calreticulin expressed on the cell surface. Normally, calreticulin is located in endoplasmic/sarcoplasmic reticulum (4), in the cell nucleus (5), and partly on the surface membrane (6). Cellular stress induces its surface expression. In this case, calreticulin acts as a pro-phagocyte signal binding to CD91 receptor on phagocytes, which leads to the absorption of the target cell. Normal cells with a low level of surface calreticulin are not destroyed because they send anti-phagocytic signals with their surface CD47 (7). Certain cancers present super-expression of surface calreticulin, but most normal cells have low calreticulin levels. Enhanced CD47 expression correlates with high calreticulin expression, and that is necessary to avoid calreticulin mediated phagocytosis (8–10).

### Heat Shock Proteins (HSP) and NK-cells

Unlike normal cells, tumor ones have an elevated expression of heat shock proteins (HSP). These proteins play a different role in the intracellular or extracellular settings. On the one hand, intracellular HSPs defend tumor cells from the stressful impact of the microenvironment (11, 12), which becomes a problem for the antitumor therapy. On the other hand, membrane and extracellular Hsp70 have a stimulating immune effect (13–15). Some HSPs can bind intracellular antigen peptides. Such peptide complex may come out on the cell surface as a result of cell lysis and other processes. APCs have surface receptors that capture the complex and engulf it. APCs can incorporate antigens linked to HSPs and on activation present these antigens to CD8+ T-cells, thus promoting cytotoxic lymphocyte activity (16, 17). Moreover, surface Hsp70 mediates cytotoxic NK functions. Surface Hsp70 was found on plasmatic membranes in different tumor cell cultures (18) and tumors of cancer patients (19), while normal tissues had no Hsp70 (20). Cytokine activated NKs recognize and lyse tumor cells with surface Hsp70 (21, 22). CD94 receptor on the NK probably participates in the Hsp70 recognition. Tumor cell surface HLA-E serves as an inhibiting signal, whereas Hsp70 is an activating signal for different complexes of CD94/NKG2D receptors on the NKs (23, 24).

### B7-H6 and NK-cells

Tumor cell surface B7-H6 is a ligand for NKp30 activating receptor on the NKs (25). Interaction of B7-H6 and NKp30 induces cytotoxic functions of IL-2 activated NKs (26, 27). Generally, B7-H6 protein has not been found in normal tissues (26), though it is expressed on the CD14+/CD16+ pro-inflammatory monocytes in sepsis (28). However, some studies occasionally detected B7-H6 by immunohistochemistry in normal tissues and showed no essential differences in B7-H6 expression between a tumor and normal tissue (29, 30). Other authors showed elevated surface B7-H6 in breast (31) and ovarian cancers (32), melanoma (33), and glioma (34), while normal tissues were negative of this parameter (34). Therefore, it seems that surface B7-H6 rate may vary with the tumor type. Some authors noted that higher expression of both surface and soluble B7-H6 in ovarian cancer was associated with the down regulation of the NK function (35). This fact may partly explain the immune system failure to recognize tumor cells with overexpressed B7-H6.

### MIC A/B, NK and T-cells

Many studies indicate NKG2D as an activating receptor that helps the immune system to distinguish tumor from normal cells. Homodimer NKG2D is expressed on all NKs as well as CD8+ αβ, γδ T-cells, and some NKT-cells (36–38). NKG2D receptor can recognize highly polymorphic stress-induced molecules MICA and MICB (major histocompatibility complex class I chain-related protein A or B) related to MHC I (39). MICA/B proteins are absent on the normal cells or a minor number of them is found on the intestinal epithelial cells (40). However, these proteins are often expressed in patients with cancer (41), such as lung carcinoma, renal, prostate, ovarian, and colon cancer (42), hepatocellular carcinoma (43), melanoma (44), and leukemia (45). MICA/B expression increased in non-tumor cell lines in various stress conditions including DNA damage (46) and viral infection (47). Moreover, NKG2D receptor can recognize other proteins expressed on the stressed cells, such as ULBP (UL16-binding proteins) (48). T-cell activation requires firstly, the signal from T-cell receptor, secondly, the co-stimulating factor CD28, substituted by NKG2D in some cases (47). MICA or MICB ligand interaction with NKG2D is a potent activating signal for NKs that can result in NK recognizing and lysing the target cell (36, 49). However, the decision of NK killing a tumor cell will be made based on the summarized effects of the activating and inhibiting receptors (50, 51).

Besides direct cytotoxicity, NK-cells can stimulate T-cell response by inducing dendritic cell maturation (52, 53). Pre-activated NKT-cells also induced DC maturation in some experimental models (54). These mechanisms facilitate the adaptive immune system to fight against the tumor. On the whole, to activate the adaptive immune system, APC should recognize the tumor and the tumor antigens should be presented to the adaptive immune cells. DCs are considered the most important APCs. DC maturation is mediated by certain cytokines produced by NK and other cells after tumor recognition and stimulated by DAMP (Damage-associated molecular patterns) released in stress and cell death. However, DAMP functions are ambiguous since they can have an antitumor effect on the one hand, and may boost tumor development on the other hand (55).

### Cancer-Testis Antigens, T-Cells

Although cancer-testis (CT) antigen expression in normal tissues of the adults is restricted to the male germ cells, CT spontaneous expression can be registered in tumor cells (56). Male germ cells lack HLA-I molecules (57); they are located at the immune privileged sites and cannot present antigens to T-cells. CT antigen expression was detected in the thymic epithelial cells that are responsible for negative selection of autoreactive T-cells (58). Nevertheless, patients with cancer often develop immune reactions to CT antigens (59), which involve both cellular and humoral responses. At present, the number of CT antigens includes over 200 protein families (60). The cancer testis database presents a lot of studies that have demonstrated immune response to these proteins with NY-ESO-1 being the most immunogenic one (60). Correlation of low functional activity of T-cells recognizing PRAME and an enhanced number of immune suppressive cells was observed in CML (61), which may explain inefficient immune response and tumor progression.

### Mutant Proteins (Neoantigens), T-Cells

Many mutations occur in the tumor as a result of its genetic instability (62, 63). Recent studies have shown that tumor antigens appearing after the mutations of normal genes are highly immunogenic. Quite a few examples demonstrate T-cell recognition of mutant proteins presented in the HLA-I context (64). On the one hand, mutations increase tumor immunogenicity, while on the other hand, they are involved in different pathways, including immunosuppression, that contribute to tumor evasion from the immune surveillance. The situation changes when immunosuppression declines due to PD-1 or CTLA-4 blockade. The studies showed that a higher mutation load of the tumor was associated with higher sensitivity to the PD-1 blocker therapy in the studied cancer types (65–68). The findings imply that tumors with a larger number of mutations were more immunogenic. The murine sarcoma model showed that mostly mutant neoantigens were responsible for recognizing the tumor during anti- PD-1 and CTLA-4 treatment (69). Personalized vaccines that induce immune response to the mutant tumor neoantigens demonstrated an effective clinical outcome though the trials involved a small number of vaccinated patients (70, 71). Therefore, the tumor has a large number of antigens to be recognized and destroyed by the immune system.

The above discussed information refers to the established tumors. However, some authors studied the immune surveillance of pre-malignant cells. Kang et al. introduced a genetic construction into the livers of mice, which activated Nras oncogene. Normal hepatocytes with the genetic construction entered the cellular senescence program, which prevented the tumor growth. The livers of these mice with senescent hepatocytes were infiltrated by immune cells, expressed pro-inflammatory cytokines, and therefore had decreased numbers of Nras-positive cells. As a result, normal mice did not develop any tumors. However, if monocytes/macrophages or CD4+T-cells, but not others, were removed, these mice developed hepatocellular carcinoma (HCC) (72). This study demonstrated the immune surveillance even at the stage of pre-malignant cells.

## Mechanisms of Immunosuppression in the Tumor Microenvironment

Despite the presence of tumor-associated antigens the immune system destroys the established tumors very rarely. Tumor microenvironment includes immunosuppressive factors as well. We assume that it is the immunosuppression which contributes most of all to the tumor evasion from the immune reactions. Mechanisms of immunosuppression in the tumor microenvironment have been studied in detail in many profound reviews (73, 74). This section presents some of those mechanisms. Part of them is generated by the tumor cells, while other mechanisms are triggered by the recruited normal cells of the tumor microenvironment. The section does not classify the mechanisms with regards to their origin. We have found just a vague design of immunosuppressive mechanisms hierarchy and classification. Therefore, firstly, we describe them in general and in the following section we will suggest a structure of the immunosuppressive cells' hierarchy.

### Surface Expression of Ligands for Immune Cell Inhibitor Receptors

As shown above, tumor cells express CD47 to defend from phagocytosis induced by calreticulin (8). Surface PD-L1 expression is frequently detected on tumor cells or on the cells of the tumor microenvironment. Binding to its PD-1 receptor PD-L1 molecule can inhibit T-cell activation (75). Besides PD-1, T-cells have other inhibiting receptors, such as LAG-3, which ligands may be expressed in tumors (76). Another inhibitor CTLA-4 is mainly expressed on regulatory T-cells (Treg) and on the activated conventional T- cells (Tconv). CTLA-4 blockers may enhance anti-tumor T-cell activity. The studies showed that CTLA-4 can reduce the number of CD80 and CD86 ligands on APCs by transendocytosis leading to the inability of APCs to activate T-cells (77, 78).

### Immunosuppressive Cells

It is now well known that quite a few of the immune cell populations can have suppressive functions and are found in the tumor microenvironment. The most substantially studied lymphoid cells are Tregs and NKT-cells type II. Tumor-associated macrophages (TAM) of M2 type and myeloid-derived suppressor cells (MDSC) are the most studied myeloid cells. Immature dendritic cells that cannot present tumor antigens have a significant impact on tumor evasion from the immune surveillance.

### Secretion of Soluble Immunosuppressive Factors

Tumor microenvironment accumulates an increased number of immunosuppressive cytokines such as TGF-β (79) and IL-10 (80). Multifunctional factors PGE2 (81), IL-6 (82), and others exert their immunosuppressive functions in the settings of tumor microenvironment. Besides, extracellular adenosine accumulates there and binds to its receptors on the immune cells, which fosters suppressor activity of the immune cells (83). Lactate presence in tumor microenvironment can stimulate immunosuppression, as well (84).

Most tumors express at least one type of NKG2D ligands and therefore they must be sensitive to NKG2D-dependent immune response. However, soluble forms of NKG2D ligands shed from the tumor cell surface and thus facilitate tumor evasion from the immune surveillance. The serum amount of soluble NKG2D ligands correlates with tumor progression in some cancer types (85).

### Exhausting the Nutrients in Tumor Microenvironment

TAM and MDSC produce arginase-1 enzyme resulting in exhausted arginine in the microenvironment. L-arginine is an amino acid needed for T-cell proliferation and ζ-chain synthesis of the T-cell receptor (TCR). Arginase-1 destroys arginine, causes TCR ζ-chain impairment, and eventually blocks activation and proliferation of T-cells (86). MDSC can exhaust L-cysteine by consumption and engulfment. This amino acid is important for T-cell activation. It is present in the form of cystine in the microenvironment. Although T-cells cannot absorb cystine, they depend on cysteine, which is produced mainly by mature dendritic cells and macrophages when they present antigens. These cells absorb cystine, split it to cysteine, and partially transfer it to T-cells. MDSCs absorb cystine but do not transfer it to T-cells (87). Tumor cells, DCs, macrophages, and MDSCs can produce immunosuppressive intracellular enzyme indolamin-2,3-dioxygenase (IDO). IDO inhibiting effect on T-cells is associated with the depletion of the essential amino acid tryptophan and formation of suppressive tryptophan metabolites as a result of this process (88). Therefore, antitumor immune responses can be inhibited by a number of mechanisms.

## Common Mechanisms for Wound Healing and Tumor Microenvironment

It has already been shown that the malignant process has some similar features with wound healing (89–91). It is therefore reasonable to search for and discuss their common mechanisms. Wound healing is a complex process that is divided into several phases. They have 3 major stages: inflammation, proliferation, and tissue remodeling. It should be noted that the definition refers mainly to skin wound healing because they were studied most intensively. To summarize the features of this process, we will use the term trauma healing.

1. Once the trauma has occurred, constriction of the blood vessels and platelet aggregation develop in order to stop bleeding. Then different inflammation related cells are recruited to the site: neutrophils are recruited at the early phase and monocyte/macrophages appear at the later phase. Inflammation reaction is triggered by various cytokines and chemokines, as well as DAMP and PAMP. Inflammatory phase is characterized by hemostasis that prevents further damage and closes the wound. The phase also includes chemotaxis and enhanced vascular permeability that helps cell migration to eliminate cellular debris and bacteria.

2. Proliferation phase develops when the wound defect is filled with granulation tissue. Fibroblasts proliferate and produce new collagens and glycosaminoglycans that promote wound stabilization. Consequently new blood vessels develop and, finally, wound edges are sealed by an immature scar.

3. Maturation phase develops when the damaged site is restored; it reaches its maximum strength and the scar is formed. If it is a skin wound, epithelization develops and the wound edges are pulled together (92, 93).

Further we will discuss some mechanisms of immunosuppression which occur at the trauma site and during tumorigenesis. However, these mechanisms are not necessarily the same in all types of wound healing.

The studies showed that mRNA-related PD-L1 expression was high in normal human organs including heart, skeletal muscles, placenta, and lungs (94). However, protein-related PD-L1 expression was not observed in healthy subjects (95), or it was low and increased with inflammation (96, 97). For instance, the studies of experimental skin inflammation showed PD-L1 expression on some cells of microvessels and keranocytes though they were not detected in healthy skin (98).

Purinergic regulation is involved in the resolution of inflammation. This system is rather complex requiring counter-regulatory mechanisms. We will describe it in a simple schematic way and it may be found in the referred review in detail (99). Normally, ATP molecules are located intracellularly and just a small number is found in the extracellular matrix. ATP is rapidly released into the extracellular matrix in case of cellular stress or cell damage. ATP has chemotactic and stimulating effect on immune cells when its high concentration accumulates in the extracellular matrix. Enzymes split ATP on the immune cell membranes to continue the proliferation phase. CD39 molecules can split ATP and ADP down to AMP. CD73 can split AMP to immunosuppressive adenosine. Adenosine binds to its receptors on a great number of immune cells and has an anti-inflammatory effect.

Lactate accumulates in wounds in some cases (100, 101). However, the data about its function are ambiguous: on the one hand, experimental addition of lactate improved wound healing (102, 103), on the other hand, high lactate concentrations have a negative effect on fibroblast and endothelial cell viability (101).

Some authors suggest that the major Treg function is the defense against autoimmune reactions. Besides, other experimental studies showed that activated Foxp3+Tregs accumulated in the skin wound site and improved its healing (104). Similar effects were observed in healing of some other organs (105).

Many authors consider that macrophages play a significant role in the wound-related processes (106, 107). Back in the 1970s it was found that macrophage depletion significantly delayed wound healing in animals (108). Similar results were obtained in the studies on genetically modified mice where it was possible to achieve specific depletion of macrophages in wounds (109, 110). The authors found that macrophage depletion was especially critical at the inflammatory or proliferation phases (110). At the early stage of wound healing infiltrating monocytes and residential macrophages are affected by pro-inflammatory cytokines, interferons, PAMP, or DAMP; they become activated and acquire mainly pro-inflammatory phenotype M1. They eliminate microorganisms by phagocytosis, remove dead cells and cellular debris and produce pro-inflammatory mediators and chemokines for additional recruitment of leukocytes. Later, macrophages shift from pro-inflammatory M1 phenotype to reparative M2 type in the healing process, and express anti-inflammatory mediators and growth factors facilitating fibroblast proliferation and angiogenesis. M1–M2 transition is of ultimate importance for inflammation resolution and shifting balance to tissue regeneration (111). It is worth looking in more detail at the mechanisms which macrophages use to make final decision of polarization to phenotype M2. So far, a number of such mechanisms have been studied (111, 112). We would like to identify potential polarization mechanisms at the trauma site. And most likely, this is not just a decrease of inflammatory mediators, RAMP and DAMP in the microenvironment, but the presence of active counter-regulatory mechanisms. It should be noted that IL-4 and IL-13 cytokines, considered necessary for alternative macrophage activation in the *in vitro* experiments, were not found in the wound microenvironment in mice *in vivo* (113). There is a mechanism associated with the elimination of apoptotic neutrophils. At an early inflammatory stage, many neutrophils are found in the wound microenvironment, which help wound cleaning. However, if they persist for long, they may damage surrounding tissues (114). Macrophages induce apoptosis in neutrophils to eliminate them from the wound (115). Afterwards, macrophages remove apoptotic neutrophils by phagocytosis (116). Interestingly, phagocytosis of neutrophils is important for macrophages polarization from pro-inflammatory M1 phenotype to reparative M2 (117, 118). However, according to the most recent data, not all neutrophils die via apoptosis at the trauma site, but many of them return to the vascular system (119). Grinberg et al. discovered a counter-regulating mechanism of restricting inflammation that functions with Toll-like receptors. Toll-like receptor (TLR) 4 ligands and adenosine A (2A) ligands switched macrophages from inflammatory M1 to angiogenic M2-like phenotype (120). Immune complexes with LPS or IL-1 mediate M2 polarization, as well (121). This may imply another type of a counter-regulating mechanism.

Though some authors noted that lactate can shift macrophage polarization to M2 in tumor microenvironment (84), we consider that the mentioned mechanism may play only a supplementary role in case of wound healing because of ambivalent lactate features. Many studies showed that PGE2 can shift macrophage phenotype to M2 (122). It is well known that PGE2 has pro-inflammatory function (at the early stages of inflammation), as well as anti-inflammatory activity (at the final stages when PGE2 mediates wound healing) (123). In this regard, there are doubts that PGE2 is an independent factor affecting macrophage polarization. Perhaps its functions are associated with other mediators currently present in the microenvironment. Therefore, it may be assumed that the transition from inflammation to proliferation requires counter-regulatory mechanisms.

Besides macrophages in the trauma site, an increased number of CD14+/HLA-DR^low/−^ monocytes were registered in the peripheral blood (124, 125). A similar increase of these cells was found in case of malignant process (126–129). The reports show that such monocytes of cancer patients have immunosuppressive functions and are referred to as MDSC (126, 127). They are less studied in case of trauma; though some data indicate that the increase in these cell numbers is associated with the risk of secondary infections (130). MDSCs were found in the trauma site in the mice studies (131). Another report showed that MDSCs supported trauma healing (132). It is highly likely that M2 macrophages and MDSCs are the same cells of different status with similar functions since MDSC in tumor microenvironment can differentiate into TAM (133). Moreover, the studies on murine models showed that monocytes accumulated in the trauma site and could present either pro-inflammatory or anti-inflammatory functions similar to those of M1/M2 macrophages (134–136). Therefore, it is not always possible to distinguish these cells, and this paper will regard monocytes, macrophages, immature DC, and monocyte-derived MDSC as a single system of myeloid cells. There is a term of mononuclear phagocytic system, but this paper will regard them as monocytes/macrophages.

When comparing wound healing with the tumor process, there arise some issues. For instance, why similar mechanisms lead to inflammation resolution in injury, but do not stop inflammation in tumors. And there are certain differences between a malignant process and inflammation caused by chronic infections (137). A vivid comparison was made for the tumor as a “non-healing wound” (89). Another definition may be “continuous immunosuppressive inflammation.” The condition looks like a frozen process at some transitional stage between inflammation and proliferation. Studying the role of stem cells in trauma healing will help better understanding of this phenomenon. Probably, the interaction between myeloid and stem cells has common characteristics with the “seed and soil” hypothesis of metastases formation (138).

Wound healing involves such important stem cells as mesenchymal stem cells (MSC), hematopoietic stem cells (HSC), adipose tissue stem cells (ADSC), and endothelial progenitor cells (EPC) (139). We will use the term “stem cell” to describe their common features or indicate a certain cell type where appropriate. It is well known that stem cells can migrate to the trauma site (139, 140). Stem cells probably can improve wound healing by two major mechanisms–by secreting mediators necessary for healing (as a result of the release of inflammatory mediators together with the key cytokines and growth factors) and by differentiation into the cell types necessary for the wound closure. However, mechanisms of stem cell action in the wound healing have not been characterized in detail, yet.

Pathologic inflammatory reaction to the trauma can disrupt stem cell functions. For instance, polymorphonuclear cells recruited to the site of injury caused necrosis of endothelial precursor cells (EPC), possibly, as a result of reactive oxygen species action (141). Therefore, it is more probable that stem cell functions of tissue reparation are realized mainly after inflammatory phase and thus, stem cells should be able to control inflammation independently. It is already well known that MSCs have immunosuppressive functions (142, 143).

Some reports demonstrate that inflammatory cytokines induce MSC immunoregulatory functions (144–146). In fact, such microenvironment is observed at the inflammatory phase of wound healing. Pro-inflammatory cytokines, toxins of infectious agents and hypoxia can stimulate MSCs to produce growth factors including epidermal growth factor (EGF), fibroblast growth factor (FGF), platelet growth factor (PDGF), transforming growth factor β (TGF-β), vascular endothelial growth factor (VEGF), hepatocyte growth factor (HGF), insulin-like growth factor-1 (IGF-1), angiopoietin-1 (Ang-1), keratinocyte growth factor (KGF), and stromal cell factor-1 (SDF-1). These growth factors consequently promote development of fibroblasts, endothelial cells, and tissue precursor cells that build up tissue regeneration and restoration (147).

Some interesting specific features of the interaction between stem and immune cells, particularly myeloid ones, are worth mentioning. Numerous experiments showed that MSCs regulate macrophage and DC functions by soluble mediators; although intercellular contacts play an important role as well (148, 149). For instance, MSCs inhibit macrophage phenotype polarization to M1 type in the animal model of sepsis (150); similar results of macrophage polarization were obtained on the rat model of trauma (151). MSCs also inhibit DC maturation (152, 153). M2 macrophages and immature DCs are usually found in the tumor microenvironment. The papers present several descriptions of mechanisms of suppressive MSC effect on myeloid cells. For example, MSCs produce PGE2 (122, 154) and interleukine-1 receptor antagonist (IL1RA) (155).

The interaction between pro-inflammatory cytokines and growth factors that may simultaneously present at the wound site during the transition process from inflammation to proliferation, which, in fact, has been poorly studied so far, is also worth being considered. That brings up a some questions: “is simultaneous presence of pro-inflammatory cytokines and growth factors in the microenvironment immunosuppressive?,” and “doesn't that give a signal for macrophage phenotype polarization to M2 type and for inflammation resolution move forward to proliferation phase?” No such investigations of wound healing have been identified, although there are some reports that partly support this possibility. Mesenchymal stem cells, derived from the umbilical cord, suppressed monocyte differentiation into DC leading to the phenotype that produced IL-10. This was the result of the MSC production of Il-6 and HGF cytokines (156). A similar study generated DCs by monocyte cultivation in the presence of IL-4 and GM-CSF. Multipotent MSCs added into the culture stopped monocyte differentiation and shifted the phenotype to produce IL-10. The effect was associated with IL-6 cytokine production by multipotent MSCs (157). It should be noted that the mentioned culture contained IL-6 and GM-CSF; and that could have had immunomodulating effect on monocytes. Huen et al. found that GM-CSF stimulated alternative macrophage activation after renal ischemic/reperfusion injury (158). GM-CSF is regarded as a pro-inflammatory factor if no additional stimuli are involved.

It is unclear whether the immunosuppressive effect of pro-inflammatory cytokines and growth factors plays an essential role in injury healing. Can it represent one of the mechanisms triggering macrophage polarization to M2 phenotype? So far, the data are insufficient to answer the question. But the described mechanism seems to be of great importance in oncology, which we will discuss below.

## Monocytes/macrophages in Tumor Process

Most authors assume that macrophages play the key role in inflammation resolution and transition to the proliferation phase in wound healing. Since the tumor involves natural mechanisms of immunosuppression, it is presumed that myeloid cells such as monocytes/macrophages (including monocytes, macrophages, immature DC, monocytic MDSC) play an essential part in these mechanisms as well. A large number of studies proved macrophage presence in the tumor microenvironment (159). TAM (160) and MDSC (129) functions in the malignant process were well described in some studies. The results of animal studies showed that macrophage (161) or MDSC (162) depletion was associated with the reduction of tumor burden.

However, the authors may have different understandings of the regulatory cell hierarchy. And most likely, T-regulatory CD4+/CD25+/FoxP3+ cells rather than monocytes/macrophages can have the key role in tumor immunosuppression. Regarding this assumption, it should be noted that adaptive immunity is activated by the signals received from the cells of the innate immunity. Treg cells function in cooperation with APCs. Most APCs are DCs and macrophages. Treg cells need antigen stimulation via APC to implement their suppressive function. In turn, Treg suppressive mechanisms function mainly as a result of their interaction with APCs decreasing APC ability to activate effector cells (77, 163). Therefore, macrophages and DCs probably regulate Treg accumulation and activation; thus Treg cells depend on these APCs. We consider that induced Tregs contribute significantly to the tumor tolerance as compared with natural (thymic) Tregs. Normal function of the induced Tregs in maintaining tolerance can be seen in the lungs and intestines. Numerous non-dangerous antigens enter the body through these organs; the reaction to such antigens may result in more harm than good. Immune tolerance to inhaled antigens in the lungs is mainly mediated by T-regulatory cells, which can inhibit effector T cells with a variety of mechanisms. The reports show that regulatory antigen-presenting cells (macrophages and DCs) are crucial for Treg generation and maintenance of the suppressive microenvironment in the lungs (164, 165). Moreover, the studies showed that DCs promote not only Treg accumulation, but, conversely, confine Treg differentiation (166). In fact, there are few reports of this kind regarding tumor microenvironment. Jitschin et al. showed Treg dependence on MDSCs *in vitro* (127). Hoechst et al. showed that monocytic MDSCs induce Treg generation *in vitro* (167, 168). The studies on tumor models found that MDSCs caused tolerance to the tumor as a result of Treg accumulation (169, 170).

In regard to the cell hierarchy, we do not insist that monocytes/macrophages have more significant suppressive effect on antitumor immunity than Tregs. Regarding the cell hierarchy, there is only a suggestion that Tregs have a dependent position in relation to monocytes/macrophages. According to these findings, myeloid cells may be a more promising therapeutic target. Thus, in case of effective targeting monocytes/macrophages, Tregs will be automatically affected as well.

## Cytokine Interaction in Tumor Microenvironment

This section discusses the impact of certain soluble factors of tumor microenvironment on the polarization of monocytes/macrophages. P53 mediates the cellular aging program, thus protecting the cell from malignant transformation (171). Lujambio et al. showed that senescent stellate cells with unmodified p53 in the liver express factors that promote macrophage polarization to M1 phenotype. These macrophages were able to attack aging cells in culture. At the same time, proliferating p53-deficient stellate cells secrete factors that stimulate macrophage polarization to M2 phenotype (172). Another study evaluated the immuno-mediated clearance of aging hepatocytes to prevent tumor development, a process also called “senescence surveillance.” The study found that “senescence surveillance” requires recruitment and maturation of CCR2+myeloid cells, while their depletion causes HCC growth. On the other hand, the tumor cells prevent maturation of recruited myeloid precursors, and, in addition, these myeloid cells become immunosuppressive (173). Besides HCC, some other cancer types affect myeloid cells in the same mode. Lechner et al. studied about 100 different tumor cell lines cultured in the presence of mononuclear cells of healthy donors. The results showed that 45 cell lines stimulated monocyte transformation into CD33+ MDSC-like cells that could inhibit T-cells (174). Similar results were obtained in the studies of CLL cell cultured with the mononuclear cells of healthy donors (127).

Naturally, the question arises: “what tumor-produced factors lead to immunosuppression of monocytes/macrophages?” Lechner et al. studied 15 immune factors of the tumor cell lines by RT-PCR. Cytokine mixtures were tested for their ability to generate suppressive CD33+ cells from healthy donor mononuclear cells *in vitro*. The combination of GM-CSF and IL-6 cytokines demonstrated the highest immunosuppressive effect, and the combinations of GM-CSF and IL-1β, PGE2, TNF-α, or VEGF showed immunosuppressive activity, as well (175). Pleiotropic IL-6 role in tumor immunosuppression (176) may be reasonably explained by interaction with other soluble factors. However, when considering GM-CSF, the situation is somewhat more complicated. The GM-CSF immunostimulating and regulatory functions have been discussed for long, but the problem still remains unresolved (177, 178). The above mentioned papers describe in detail the controversial issues related to the problem, however, they propose only their own subjective opinion. The issue is complicated; a number of studies received the opposite results when cultivation of myeloid precursors with GM-CSF led to tolerogenicity (179, 180) or DC maturation (181). However, some authors tried to explain the controversial effects of GM-CSF by its different concentrations. Progenitor cells derived from bone marrow treated with a low dose of GM-CSF may develop into tolerant immature DCs, while the same cells treated with a higher dose of GM-CSF may develop into a mixture of mature and immature DCs (182). This phenomenon can be explained by the fact that the culture with DC precursors could have included low concentrations of pro-inflammatory cytokines, and after addition of GM-CSF in a low dose, they had a joint immunosuppressive effect. In case of GM-CSF high doses, the difference between GM-CSF and pro-inflammatory cytokine concentrations was far more significant, and therefore GM-CSF manifested its immunostimulating effect. On the other hand, there are some *in vitro* studies where GM-CSF had a suppressive effect in high concentrations, and it is more difficult to explain that phenomenon. Though, it should be noted that the latter studies had certain differences in the methodology as compared with the studies where GM-CSF showed a pro-inflammatory effect (181, 183). Nevertheless, Marigo et al. failed to generate immunosuppressive myeloid cells when cultured with GM-CSF only, but received them in the culture with a combination of GM-CSF + IL-6 (184). Similar results were achieved in some other studies. Immunosuppressive MDSC were obtained *in vitro* with combinations of such cytokines as GM-CSF + IL-6 (185–187), GM-CSF + IL-6 + PGE2 (188), GM-CSF + IL-6 + G-CSF (189), PGE2 + GM-CSF + IL-4 (190), GM-CSF + IL-6 + IL-1β (191). Cytokine combination IL-6 + G-CSF inhibited differentiation and activation of dendritic cells (192). At this point it is worth remembering the suppressive effect of mesenchymal stem cells on monocytes, which decreased after the blockade of some pro-inflammatory cytokines and growth factors, as it was described above in the section on wound healing.

Furthermore, one may ask the question: “is there such a combination of cytokines in the tumor microenvironment?” It is now assumed that tumors are often associated with persistent unresolved inflammation; therefore, pro-inflammatory cytokines are found in the tumor microenvironment. A thoroughly studied HCC is a good example. We described above the fact that HCC development is normally prevented by inflammation and macrophages with “senescence surveillance” (75), but myeloid cells of the tumor microenvironment become immunosuppressive in the established HCC (173). Besides inflammation, a certain number of growth factors appear in the HCC microenvironment. Later, we will talk about the results of patients' tumor studies. M-CSF high expression and increased macrophage distribution in peritumoral region was associated with HCC progression (193). The enhanced circulating TGF-β1 concentration was associated with the worse survival rate of patients with HCC (194). Serum VEGF levels in patients with HCC were significantly higher than those of healthy donors (195). FGF19 expression correlated with tumor progression and worse prognosis in HCC (196). High serum HGF levels in patients with HCC were associated with poor prognosis after liver resection (197). Pancreatic cancer is also associated with inflammation. Two experimental studies of pancreatic cancer detected GM-CSF in patients' tumor samples after immunohistochemical staining (179, 180). Another review (198) showed the important role of inflammatory cytokines and TAM in the development and progression of pancreatic cancer and found growth factors in the tumor microenvironment. Multiple myeloma is an example of hematological malignancies, which includes the enhanced rate of some growth and pro-inflammatory factors (199–201). The studies found that HGF, bFGF and G-CSF expressions in head and neck squamous cell cancer were negative prognostic factors for patient survival (202). The studies of breast cancer found cytokine G-CSF, IL-6 and IL-17 expression in the serum of cancer patients, but not of healthy volunteers (203). The study of serum cytokines in melanoma reported that the concentrations of several factors (IL-1α, IL-1β, IL-6, IL-8, IL-12p40, IL-13, G-CSF, MCP-1, MIP-1α, MIP-1β, IFN-α, TNF-α, EGF, VEGF, and TNF-RII) were significantly higher in patients with high-risk resected melanoma compared to those of healthy donors. In addition, IFN-α-2 therapy led to a significant decrease in the levels of growth factors, such as VEGF, EGF, and HGF (204). Another paper reviews the evaluation of serum cytokine profile in patients with several cancer types (205). Although it pays less attention to growth factors but it shows their presence. It is worth mentioning that the concentration of cytokines directly in the tumor microenvironment should be regarded as of higher importance.

**Figure 1:**
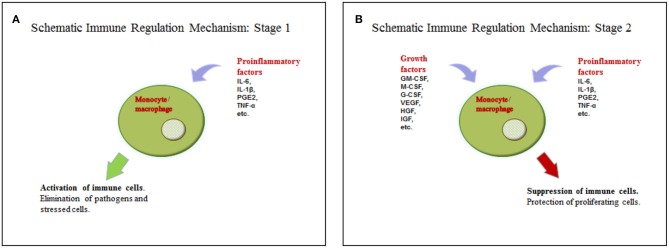
**(A)** Activation of the immune cells by pro-inflammatory cytokines. **(B)** Suppression of the immune cells by the combination of pro-inflammatory cytokines and growth factors.

We suggest a hypothesis with regards to the above discussed data that may explain such functions of soluble factors in tumor microenvironments. According to the hypothesis, a tumor cell acquires some characteristics of a stem cell, including the ability to regulate immunity in a similar way to the interaction between mesenchymal stem cells and macrophages, as it is described in the section on wound healing. Yet, in case of injury, MSCs are found in the inflammatory microenvironment, but they do not produce it. This location stimulates them to produce growth factors. The combination of soluble mediators (pro-inflammatory cytokines and growth factors) promotes monocyte/macrophage immunosuppressive activity. The process in wound healing is most likely a short-term one and helps the transition from the inflammatory to proliferation phase. Tumor cells are supposed to produce or support growth factors and pro-inflammatory cytokines in an independent manner for a long time, using this mechanism to avoid immune reactions (Figures 1A,B, Table 1). However, it is difficult to identify the necessary ratio of growth factors and pro-inflammatory cytokines that leads to immunosuppression; it may be 1:1 or even 1:5. That should be determined in the experiments. It is yet unknown whether all pro-inflammatory and growth factors have such effect. Most likely, these are IL-6 and GM-CSF. It is also important to emphasize that immunosuppression results from the combination of growth factors and pro-inflammatory cytokines, but not from the dominating immunosuppressive growth factors, such as TGF-β. Therefore, it is counter-regulating mechanism that requires two types of signaling. Undoubtedly, besides this mechanism tumor immunosuppression involves other ones. But its role is obviously underrated, and it has the potential of becoming a therapeutic target.

**Table 1:** Potential common mechanism of wound healing and tumor microenvironment.

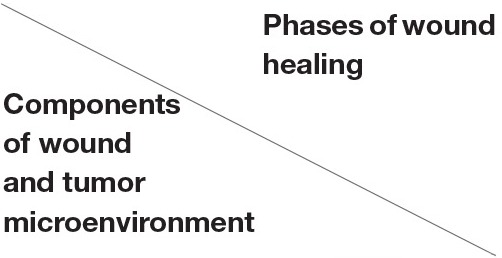	**Inflammation**	**Potential intermediate stage**	**Proliferation**
Soluble factors in the microenvironment of monocytes/macrophages	Domination of pro-inflammatory cytokines (acute inflammation). As a result, MSCs start producing growth factors.	Accumulation of pro-inflammatory cytokines and growth factors.	Inflammation resolution.
Polarization of monocytes/macrophages	M1–like phenotype	Monocytes/macrophages acquire immunosuppressive phenotype. Transition from M1 to M2.	M2–like phenotype
Similar microenvironment in tumors	Rare	Very often	Occasionally

The studies showed that different strategies of resolving tumor-associated inflammation can adversely affect tumor growth and development (206, 207). Yet, assuming the decrease of inflammation should reduce immune functions seems controversial. Our hypothesis explains such effects of reduced inflammation by the alteration of cytokine balance.

Though unexpected, elevated levels of growth factors and pro-inflammatory cytokines were observed in some autoimmune diseases. However, the processes associated with these diseases are opposite–not decrease, but excessive activation of the immune functions–and it is difficult to detect the exact cause of such processes. We suggest there might be mechanisms that are hierarchically higher than immunosuppression caused by the combined effects of inflammatory cytokines and growth factors, such as some super-antigens, and these mechanisms can block immunosuppression.

## Signaling Molecules Mediating Monocyte/Macrophage Polarization to the Immunosuppressive Phenotype

The characteristics of the signaling pathways promoting monocyte/macrophage immunosuppression are far from being complete, though some data are already available. A more detailed study of signaling could provide additional data for understanding our hypothesis. At the initial stages, the signal transmission from the receptors of growth factors and pro-inflammatory cytokines is achieved with the “integrated” tyrosine kinases, Jak-STAT, MyD88, TRAF, etc. Understanding the process is rather difficult because of the fact that growth factors such as EGF, PDGF, VEGF, M-CSF use “integrated” tyrosine kinases, whereas colony-stimulating factors such as GM-CSF and some pro-inflammatory cytokines, including IL-6, use Jak–Stat signaling. Therefore, it is difficult to identify any regular patterns at the initial stages of the signaling pathways of growth factors and pro-inflammatory cytokines.

Cytokine IL-6, which has a dual role in the anti-tumor immunity, activates signaling proteins Stat1 and Stat3 in addition to its other functions. Stat1 is known for its anti-tumor activity, whereas Stat3 is known for promoting tumor progression and immunosuppression (208). The balance between the opposite effects of Stat1 and Stat3 is considered to be one of the mechanisms regulating the inflammatory status of macrophages. Some authors believe that Stat3 activation is the key factor responsible for the tolerance associated with tumor escape from the immune surveillance (209, 210).

Transcription factor C/Ebpß plays an important role in the differentiation of myeloid precursors into functional MDSC (184). Moreover, C/Ebpß expression in myeloid precursors was associated with immunosuppression in the murine model of sepsis (211). Other studies demonstrated some correlation between Stat3 and C/EBPβ expression in MDSC in sepsis (212) and in granulocytes during “emergency” granulopoiesis (213). Kaneda et al. report that phosphoinositide-3-kinase γ (PI3Kγ) controls the transition between suppressive and pro-inflammatory macrophages in inflammation and tumor microenvironment. Signal transduction through Akt PI3Kγ and mTor signaling pathways inhibits NFκB activation and stimulates C/EBPβ activation, thereby inducing transcriptional programs that contribute to immune suppression during inflammation and tumor growth (214). Earlier, Chen et al. found that mTOR pathway is an important element in the regulation of monocyte differentiation into TAM (215).

Some signal proteins may be mentioned, which are presumably less likely to participate in immunosuppression associated with pro-inflammatory cytokines and growth factors. A number of studies presented many details of NFkB role in TAM polarization (216), and sometimes they are controversial (217). We will describe only some of the main issues. It is considered that NFkB dimer consisting of P65/P50 subunits plays a pro-inflammatory role, while the dimer comprising P50/P50 (NFκB1, inactive status) plays an anti-inflammatory role in the immune system. Panzer et al. showed NFkB1 role in the resolution of renal inflammation. After induction of immune glomerular injury in rats, mostly NF-kB P65/P50 heterodimer complexes moved to the cell nucleus, while after inflammation resolution mainly P50/P50 homodimers were found in the cell nucleus (218). Enhanced P50/P50 expression supported pro-tumor M2 phenotype of macrophages, and blocked polarization toward M1 (219, 220). Strauss et al. found that protein RORC1/RORγ promotes TAM and MDSC formation during “emergency” granulomonocytopoiesis in cancer (221). Pello et al. demonstrated that c-Myc transcriptional factor is necessary for macrophage polarization to M2 phenotype (222). A number of studies reported that IRF 4 transcription factor (interferon regulatory factor) can participate in an alternative macrophage activation (223, 224).

## Conclusion

The paper summarizes the available data on the tumor interaction with the immune system. Cell stress and mutations result in emerging antigens that make a difference between a tumor and a normal cell. Such antigens could have become the targets for immune system recognition. This makes it extremely important for the tumor to have immunosuppressive mechanisms. It is presumed that the tumor does not develop any new mechanisms for inhibiting immune reactions but uses the existing normal mechanisms. Therefore, we made an attempt to draw analogies of immunosuppressive mechanisms in the tumor microenvironment and in wound healing. At the same time, we outlined some common features and regular patterns of the microenvironment, which we put as the basis for our hypothesis. Wound healing is characterized by the simultaneous presence of growth factors and pro-inflammatory cytokines in the MSC microenvironment during the transition stage from inflammation to proliferation. We suggest that these cytokines function in cooperation and thus have a regulatory effect on monocytes/macrophages. The affected monocytes/macrophages transfer the immunosuppressive pattern onto the cells of the adaptive immune system. The above presented data demonstrate that such effects of pro-inflammatory cytokines and growth factors can be used by tumor cells to evade immune surveillance. The described details help to explain the phenomenon of the immunosuppressive inflammation in the tumor microenvironment. The combined effect of growth factors and pro-inflammatory cytokines on monocytes/macrophages has been poorly studied, yet. In case if this hypothesis is proved, the cytokine interaction could become a promising therapeutic target and have a very wide range of applications, both in oncology and treatment of some other conditions associated with abnormalities in the immune system.

## Author Contributions

AP wrote the manuscript. IS critically reviewed the manuscript and contributed to the figures. All authors agree to be accountable for the content of the work.

### Conflict of Interest

The authors declare that the research was conducted in the absence of any commercial or financial relationships that could be construed as a potential conflict of interest.
